# Revisiting magnetic resonance imaging pattern of Krabbe disease – Lessons from an Indian cohort

**DOI:** 10.25259/JCIS-18-2019

**Published:** 2019-05-24

**Authors:** Karthik Muthusamy, Sniya Valsa Sudhakar, Maya Thomas, Sangeetha Yoganathan, Christhunesa S. Christudass, Mahalakshmi Chandran, Hirenkumar Panwala, Sridhar Gibikote

**Affiliations:** 1 Departments of Neurological Sciences, Christian Medical College, Vellore, Tamil Nadu, India.; 2 Departments of Radiodiagnosis, Christian Medical College, Vellore, Tamil Nadu, India.

**Keywords:** Dentate hilum, Krabbe disease, Magnetic resonance imaging, Optic nerve hypertrophy, Tigroid pattern

## Abstract

**Context:**

Krabbe disease shows considerable heterogeneity in clinical features and disease progression. Imaging phenotypes are equally heterogeneous but show distinct age-based patterns. It is important for radiologists to be familiar with the imaging spectrum to substantially contribute toward early diagnosis, prognostication, and therapeutic decisions.

**Aims:**

The study aims to describe different magnetic resonance imaging (MRI) patterns observed in a cohort of children with Krabbe disease and to assess correlation with age-based clinical phenotypes.

**Materials and Methods:**

This is a retrospective descriptive study done at the Departments of Radiodiagnosis and Neurological Sciences of our institution, a tertiary care hospital in Southern India. Imaging features of children diagnosed with Krabbe disease over a 10-year period (2009–2018) were collected and analyzed.

**Results:**

A total of 38 MRI brain studies from 27 patients were analyzed. Four distinct MRI patterns were recognizable among the different clinical subtypes. All patients from the early and late infantile group showed deep cerebral and cerebellar white matter and dentate hilum involvement. Optic nerve thickening was, however, more common in the former group. Adult-onset subtype showed isolated involvement of corticospinal tract, posterior periventricular white matter, and callosal splenium with the absence of other supra- and infra-tentorial findings. Juvenile subgroup showed heterogeneous mixed pattern with 78% showing adult subtype pattern and 22% showing patchy involvement of deep cerebral white matter with dentate hilum signal changes.

**Conclusion:**

Krabbe disease shows distinct imaging features which correspond to different clinical age-based subtypes. This article reemphasizes these distinct imaging phenotypes, highlights a novel imaging appearance in juvenile Krabbe, and also alludes to the rare variant of saposin deficiency. Awareness of these patterns is essential in suggesting the appropriate diagnosis and guiding conclusive diagnostic workup. Large multicenter longitudinal studies are needed to further define the role of imaging in predicting the clinical course and thus to guide therapeutic options.

## INTRODUCTION

Krabbe disease (globoid cell leukodystrophy) is a rare autosomal recessive lysosomal storage disorder caused by a deficiency of galactocerebrosidase (galactosylceramidase) enzyme or its activator protein saposin. It manifests as progressive demyelination of the central nervous system with variable peripheral nerve involvement. Almost 100 years after its initial description, diagnosis and management often remain a challenge. Factors such as inherent heterogeneity in clinical and imaging phenotypes, rarity of the disease, and reduced awareness contribute to underrecognition and misdiagnosis on initial imaging studies. There is a lack for correlation between enzymatic levels and the phenotype; genotype-phenotype correlations are also heterogeneous.^[1]^ This affects the management decisions of children found positive on newborn screening. These are confounding factors in deciding who should undergo bone marrow transplant given the considerable associated morbidity. A deeper understanding of imaging phenotype, its natural evolution, correlation with prognosis, and whether imaging abnormalities predate symptoms will determine whether it is a potential biomarker and solution for the diagnostic and management conundrums. This article aims to revisit the distinct imaging patterns of Krabbe disease and attempts to correlate with age of onset-based clinical subtypes.

## MATERIALS AND METHODS

This retrospective study was approved by the Institutional Review Board, after which a comprehensive list of patients diagnosed with Krabbe disease was obtained from retrospective chart review and radiology information system. Diagnosis was based on low or undetectable enzyme levels (<10% residual activity) and/or genetic confirmation in correlation with clinicoradiological features.

Considering the fact that some cases had imaging done outside the institution and on different scanners within the institution, only those with optimal magnetic resonance imaging (MRI) brain imaging with T2, T2 fluid-attenuated inversion recovery (FLAIR), and T1 sequences of diagnostic quality were included. Serial imaging when available was analyzed for temporal changes. A pediatric neuroradiologist analyzed these images according to the pre-determined reading template. The radiologist was blinded to the age of onset of clinical symptoms but had access to the age of the patient at imaging since this was crucial in image interpretation. Pattern of supratentorial and infratentorial white matter involvement was assessed on all available sequences. Basal ganglia, thalamus, and dentate nucleus were separately assessed.

The optic nerve was considered as enlarged when the prechiasmatic segment measured more than 4 mm perpendicular to the long axis on T2, T2 FLAIR, and T1 images.^[2]^ Global or lobar cerebral volume loss was assessed and grouped subjectively into severe, moderate, and mild groups. Brain stem and cerebellar volume loss and additional incidental findings were also assessed. Computed tomography (CT), diffusion-weighted imaging, post-contrast studies, MR spectroscopy (MRS), and spine imaging were analyzed whenever available.

Based on the age at onset of symptoms, patients were further subgrouped as per Loonen *et al.* (early infantile [EI]: <6 months of age, late infantile [LI]: 7 months–3 years, juvenile [J]: 3–8 years, and adult [A]: >9 years).^[3]^ MR features were then correlated with these age-based clinical subtypes. In view of the expected small sample size, statistical analysis was restricted to descriptive statistics using SPSS16 software.

## RESULTS

**Graph 1:**
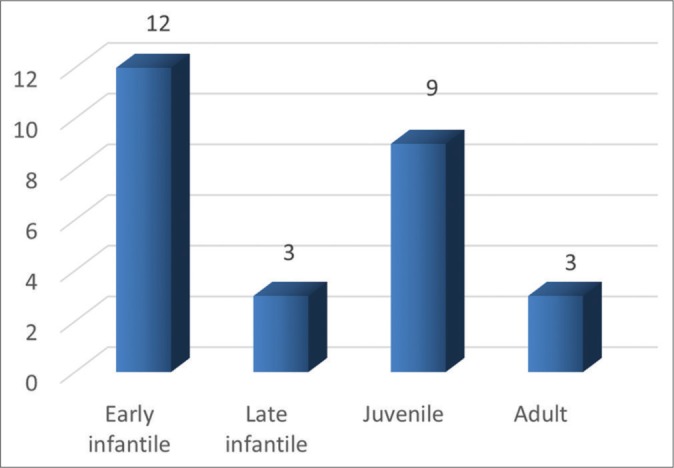
Categorization of patients by age-based clinical phenotypes. X-axis: Categorization of patients by age-based clincial phenotypes, Y-axis: Number of patients.

Twenty-seven patients (8 females and 19 males) were included. In 25 children, diagnosis was confirmed by low beta-galactocerebrosidase activity, of which four had further genetic confirmation. Two children with normal enzyme levels were genetically confirmed as saposin deficiency-related Krabbe disease. Mean age at MRI was 7 years with a range of 5 months–38 years. The number of cases under each age-based subtype is given in Graph 1.

### Imaging characteristics

Twenty-seven patients had a total of 38 MRIs. There were 14 MRIs from EI group, 4 from late infantile group, 13 from juvenile group, and 7 from adult group.

**Figure 1:**
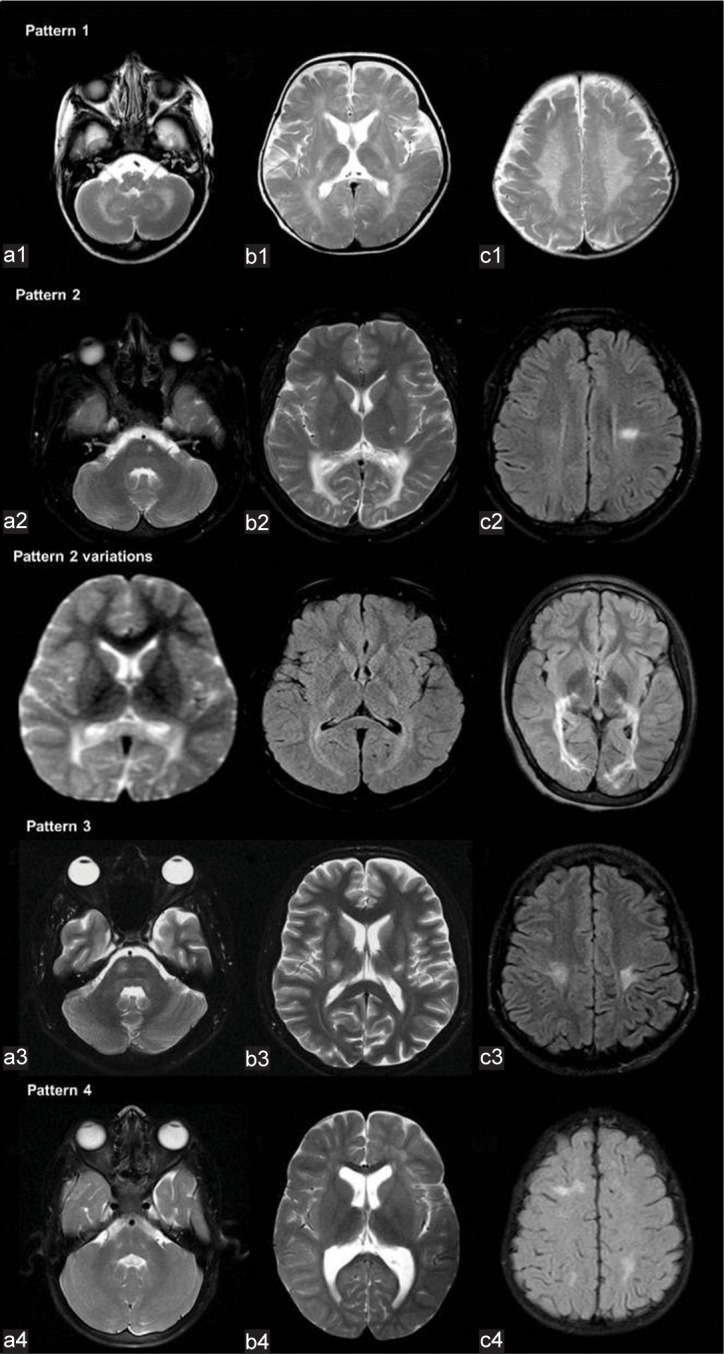
Patterns of white matter involvement in Krabbe disease. The pattern of confluent deep cerebral and cerebellar white matter signal changes along with posterior limb of internal capsule and brainstem corticospinal tracts (CSTs) involvement (a1, b1, c1) was considered as pattern 1. Confluent posterior deep white matter hyperintensity with or without CST and/or splenial involvement (a2, b2, c2, pattern variations) were considered as pattern 2. Isolated CST involvement (a3, b3, c3) was considered as pattern 3. Patchy supratentorial white matter and dentate hilum hyperintensities without deep cerebellar white matter involvement (a4, b4, c4) was classified as pattern 4.

Four patterns of white matter involvement were apparent after preliminary review [Figure 1]. The pattern of confluent deep cerebral and cerebellar white matter signal changes along with posterior limb of internal capsule (PLIC), and brainstem corticospinal tract (CST) involvement was considered as pattern 1. Confluent posterior deep white matter hyperintensity with or without CST and/or splenial involvement was considered as pattern 2. Isolated CST involvement was considered as pattern 3. Patchy supratentorial white matter and dentate hilum hyperintensities without deep cerebellar white matter involvement were classified as pattern 4.

Imaging patterns were correlated with age of onset-based clinical subtypes [Table 1]. All children (100%) with EI and LI had pattern 1. Juvenile subgroup showed heterogeneous distribution across the patterns with 66.6% showing pattern 2, 11% showing pattern 3, and 22.2% showing pattern 4. In adult subgroup, 66.6% showed pattern 2 and the rest were pattern 3.

**Table 1:** Distribution of white matter patterns among age based subtypes.

Number of cases based on age at onset	Early infantile	Late infantile	Juvenile	Adult
Pattern 1	12	3	-	-
Pattern 2	-	-	6	2
Pattern 3	-	-	1	1
Pattern 4	-	-	2	-
Total	12	3	9	3

**Graph 2:**
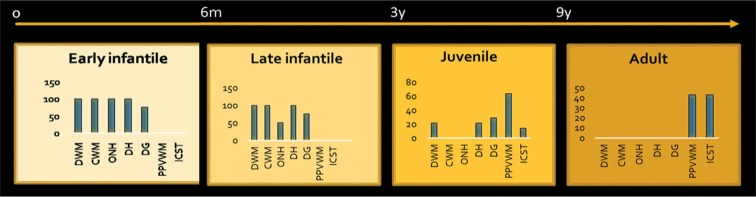
Imaging manifestations of age based subgroups. DWM: Deep white matter, CWM: Cerebellar white matter, ONH: Optic nerve hypertrophy, DH: Dentate hilum hyperintensity, DG: Deep gray, PPVWM: Posterior periventricular and deep white matter, ICST: Isolated corticospinal tract involvement. X-axis: Imaging findings, Y-axis: Percentages of involvement.

A detailed description of imaging features of different patterns and age-based subtypes is given in Table 2 and Graph 2.

**Table 2:** Imaging features in different patterns of Krabbe disease.

Pattern	Number of MRIs	Confluent deep and periventricular T2W hyperintensity with PLIC and brainstem CST involvement	Non-confluent deep and periventricular T2W hyperintensity with PLIC involvement	Isolated corticospinal tract T2W hyperintensity	Isolated posterior periventricular T2W hyperintensity with (+)/without (.) corticospinal tract involvement	Corpus callosal involvement	Thalamic volume loss and T2W hypo/hyperintensity	Confluent cerebellar white matter T2W hyperintensity	Dentate hilum T2W hyperintensity	Basal ganglia atrophy/hyperintensity	Optic nerve thickening	Cerebral atrophy	Cerebellar atrophy	Brainstem atrophy midbrain (M), pons (P)
I	18	18	0	0	0	12^a^ 2^c^ 12^d^	13^f^ 15^h^ 4^g^	18	18	Caudate atrophy-8 Caudate hyperintensity -1 Putamen hypointensity -3	16	7 s 7 mo 2 mil 2 n	4 mil	M s-8 M mil-9 M n-1 P s-9 P mil-8 P n-1
II	12	0	0	0	11 + 1-	9^a^	0	0	0	0	0	Parietofrontal atrophy-3	0	0
III	5	0	0	5	0	5^e^	1^h,g^	0	0	0	0	5 perirolandic	0	0
IV	3	0	2	0	0	2^b^ 1^a^	3^f,h^	0	3	0	0	1 mil	1 mil	0

Corpus Callosal Involvement: ^a^Diffuse splenial hyper intensity, ^b^Focal splenial hyper intensity, ^c^Genu hyper intensity, ^d^Diffuse atrophy, ^e^Focal posterior body atrophy, Thalamus: ^f^Hypo intensity, ^g^Hyperintensity, ^h^Atrophy, cerebral/cerebellar/brainstem atrophy s-severe, mo-Moderate, mil- Mild, n-No atrophy. PLIC: Posterior limb of internal capsule, CST: Corticospinal tract

### White matter

#### Pattern 1

**Figure 2:**
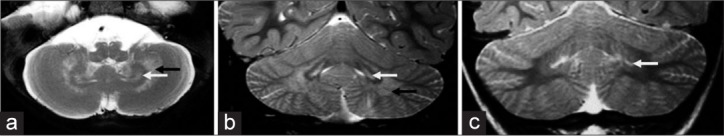
Patterns of dentate nucleus involvement in Krabbe disease. A 10-month-old boy with early infantile onset Krabbe disease -T2 axial and T2 coronal images (a, b) of shows sandwich appearance of dentate nucleus with hyperintensity of dentate hilum and surrounding cerebellar white matter. A 5-year-old girl of Juvenile subtype - T2 coronal image (c) showing isolated dentate hilum hyperintensity.

**Figure 3:**
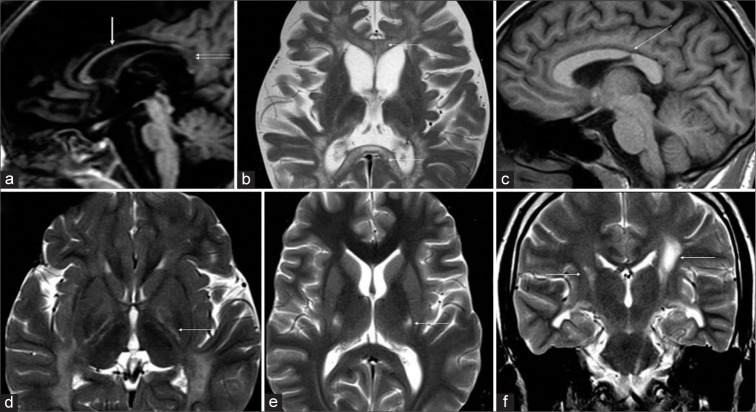
Patterns of involvement of corpus callosum (a-c) and posterior limb of internal capsule (PLIC) (d-f) in Krabbe disease. T1 sagittal (a) shows diffuse thinning of the corpus callosum (white arrow) with signal changes of posterior body and splenium (double arrows). T2 axial (b) image in a child with early infantile onset shows diffuse involvement of genu and splenium with tigroid pattern (white arrows). T1 sagittal image (c) in an adult subtype demonstrates the typical focal narrowing of an isthmic region of the corpus callosum (white arrow). T2 axial image (d) shows trilaminar appearance of PLIC (white arrow). T2 axial image (e) in adult-onset subtype shows symmetric involvement of middle-third of PLIC (white arrow) and T2 coronal image (f) shows asymmetrical involvement of the corticospinal tracts (white arrows).

Pattern 1 was seen in 18 MRIs with T2 hyperintense signal changes of deep and periventricular white matter, PLIC, CST in the brainstem, deep cerebellar white matter, and hilum of dentate nucleus [Figure 2]. Tigroid pattern was seen in supratentorial distribution in 13 MRIs, 4 of which showed additional cerebellar involvement. Diffuse thinning of the corpus callosum was seen in 12 MRIs. Splenial hyperintensity was seen in 12 MRIs and genu was involved in 2. PLIC showed trilaminar/tram-track pattern in 10 MRIs [Figure 3]. Diffuse moderate-to-severe cerebral atrophy was seen in 14 MRIs, all of which had EI onset. Four MRIs from three children with late infantile onset showed mild or no cerebral atrophy. Mild diffuse cerebellar atrophy was seen in four MRIs of EI subgroup. Atrophy of midbrain and pons was common and correlated with the degree of cerebral volume loss.

#### Pattern 2

Eight patients(6 juvenile and 2 of adult onset) with 12 MRIs belonged to pattern 2; all MRIs had confluent posterior periventricular and deep white matter signal changes. Splenial involvement was common and seen in 9 MRIs. Three MRIs from juvenile onset type did not show splenial involvement. CST and PLIC were involved in all except one from juvenile group. One case showed extensive cystic changes within the involved white matter. None had trilaminar pattern of PLIC involvement. Cerebellar white matter and dentate hilum involvement were also notably absent. Mild parietofrontal volume loss was seen in 3 MRIs of juvenile subgroup.

#### Pattern 3

Isolated CST involvement was seen in 5 MRIs belonging to 2 patients – one of juvenile and one of adult onset. The former showed symmetric CST involvement while adult case had left-sided asymmetric CST involvement on initial MRI evolving to bilateral pattern over 3-year period. Focal thinning of callosum along the posterior body/isthmus was seen in both [Figure 3]. The involved PLIC did not show trilaminar pattern. Mild focal perirolandic volume loss was seen in all MRIs.

#### Pattern 4

Three MRIs from two patients of juvenile group (siblings) showed patchy non-confluent deep white matter signal changes in frontal and posterior distribution without CST/cerebellar white matter involvement. Mild diffuse hyperintensity of PLIC and dentate hilum [Figure 2] was seen in all. Callosum showed focal central splenial hyperintensity. One of them showed remarkable interval progression of supratentorial white matter changes without cerebellar white matter involvement.

### Thalamus [Figure 4]

**Figure 4:**
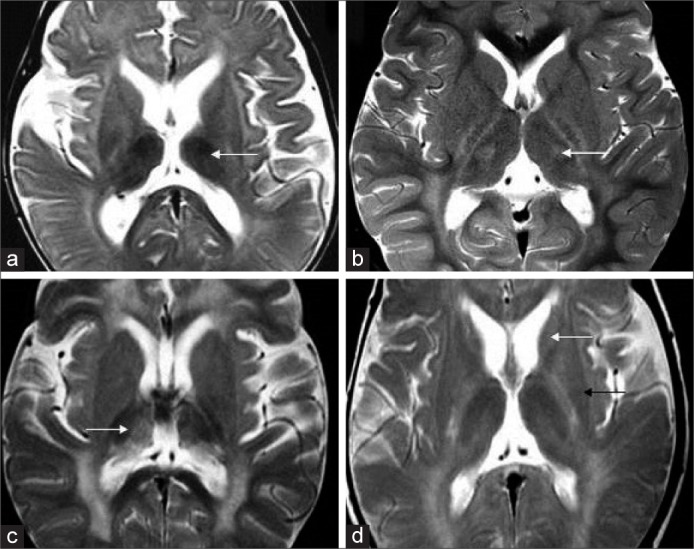
Thalamus and basal ganglia findings in Krabbe disease. Axial T2 (a) shows bilateral symmetric thalamic volume loss with T2 hypointensity in early infantile subgroup (white arrow).T2 axial in another case (b) shows bilateral ventrolateral thalamic hyperintensity (white arrow). T2 axial image (c) in yet another case shows more heterogeneous appearance with hypointensity and hyperintensity along medial apsect. T2 axial image (d) shows volume loss and hyperintensity of caudate head (white arrow) and putaminal volume loss with posterior hypointensity (black arrow).

Of 38 MRIs, diffuse thalamic hypointensity was seen in 16 MRIs (EI – 11, LI – 2, and J – 3) and heterogeneous T2W hyperintensity was seen in 5 MRIs (EI – 2, LI – 2, and J – 1). Thalamic volume loss was appreciable in 19 MRIs (EI – 14, LI – 2, and J – 3).

### Basal ganglia [Figure 4]

Caudate atrophy was seen in 8 MRIs of EI group, while one MRI showed associated hyperintensity. Three MRIs also showed putaminal hypointensity. None of the other subgroups showed abnormalities of basal ganglia.

### Optic nerve [Figure 5]

**Figure 5:**
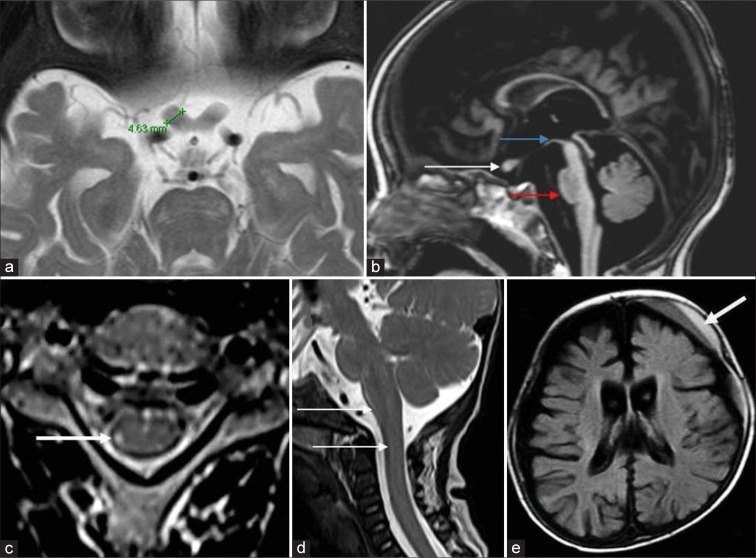
Optic nerves, brainstem, cord, and miscellaneous findings in Krabbe disease. A 7-month-old boy with early infantile Krabbe disease - T2 axial image (a) shows thickening of the prechiasmatic optic nerve which measures 4.6 mm. T1 sagittal image (b) shows the thickened optic nerve (white arrow) and moderate volume loss of midbrain (blue arrow) with hummingbird morphology and significant pontine volume loss (red arrow), axial T2 images of cervical cord in an adolescent onset case show hyperintensity of the lateral spinothalamic tract (white arrow). T2 sagittal image (d) in an early infantile onset subtype shows enlargement of cervical cord and lower brainstem (white arrows). Axial fluid-attenuated inversion recovery image (e) shows left frontal subdural collection with layered appearance (white arrow) with diffuse cerebral atrophy.

Thickening was seen in 16 MRIs of pattern 1 (EI – 14 and LI – 2). Two cases which showed normal appearance belonged to late infantile group. Optic nerve involvement was absent in all other groups.

**Spine imaging** was available in 12. Abnormal signal changes along the lateral spinothalamic tract were seen in two MRIs from a single patient in the adult group. One case from EI group showed cervical cord and lower brainstem enlargement [Figure 5].

**Figure 6:**
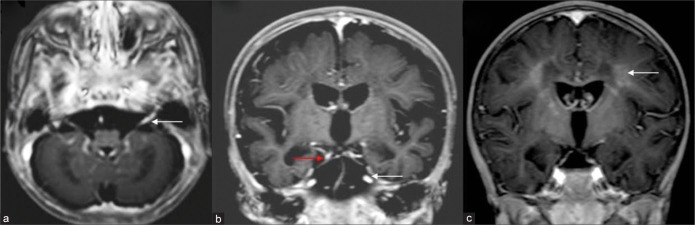
Cranial nerve and white matter enhancement. T 1 axial post-contrast image (a) shows bilateral thickening and enhancement of eighth nerve (white arrow). T1 post-contrast coronal image (b) shows enhancement along third (red arrow) and fifth nerves (white arrow) bilaterally. T1 coronal post-contrast image (c) in another child shows patchy deep white matter enhancement (white arrow).

**Post-contrast study with gadolinium** was available in 12. Three MRIs from EI group showed extensive enhancement of cranial nerves (3, 5, 7, and 8) and cervical roots. One MRI from late infantile group had similar findings along with patchy white matter enhancement [Figure 6].

### Diffusion

**Figure 7:**
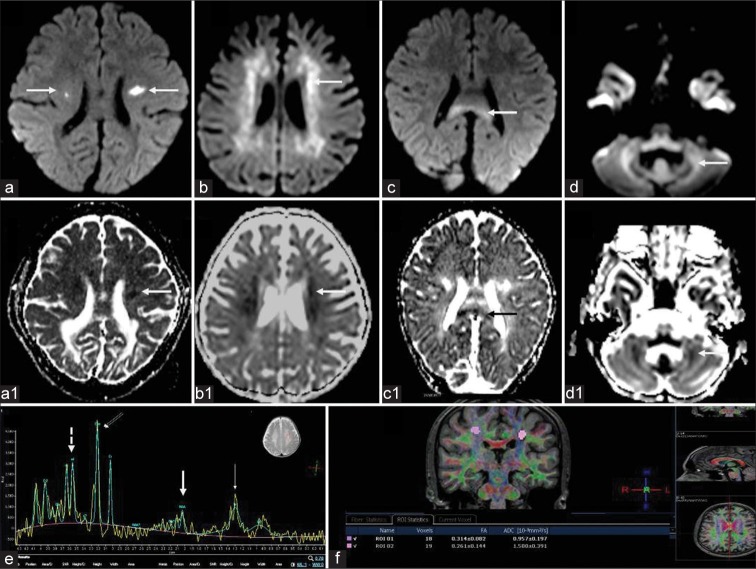
Diffusion-weighted imaging (DWI), magnetic resonance spectroscopy, and diffusion tensor imaging (DTI) findings in Krabbe disease. DWI (a-d) and corresponding ADC maps (a1-d1) in different cases show asymmetric restricted diffusion of corticospinal tracts (CSTs) (a) periventricular white matter (b), callosal splenium (c), and cerebellar white matter (d). Magnetic resonance spectroscopy from deep white matter at TE 135 (e) shows N-acetyl aspartate reduction (thick arrow) small lipid peak(thin arrow) and relative choline (double arrows) and myoinositol (dashed arrow) elevation. DTI from CSTs (f) in a case of adult-onset subtype shows reduced fractional anisotropy values.

Restricted diffusion was seen in supratentorial white matter in 3 MRIs, along with CSTs in 8 and splenium in 1 [Figure 7].

### Diffusion tensor imaging (DTI)

Only one case of adult onset had DTI (spin-echo single-shot EPI sequence with 32 gradient directions acquired on Philips Achieva 1.5 T Scanner and post-processed on MR systems Achieva) which showed a reduction in the fractional anisotropy values along the CSTs bilaterally [Figure 7].

### MRS

MRS was available in 21 at short and intermediate TE (31 and 144) and showed abnormal peaks in 13. All showed NAA reduction with additional small lipid peaks in 5 and MI peak in one [Figure 7].

### Susceptibility-weighted imaging

No gradient signal changes were appreciable in this series.

### Others

Subdural collections were seen in 3 children (EI: 2 and LI: 1) [Figure 5].

### CT brain

**Figure 8:**
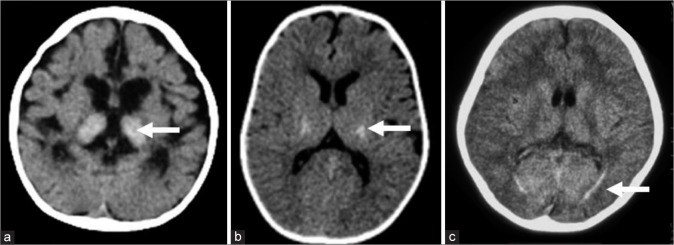
Computed tomography (CT) findings in Krabbe disease. A 9–month- old girl with progressive neuroregression diagnosed as early infantile Krabbe disease - axial CT (a) shows bilateral thalamic hyperdensity (white arrow) with significant cerebral volume loss. Axial CT image in a case with juvenile onset (b) shows bilateral symmetric hyperdensity along posterior limb of internal capsule (white arrow). Axial CT in an adult-onset case (c) depicts hyperdensity (white arrow) along optic radiations bilaterally.

CT brain was available in 11, of which 7 were abnormal. 6 showed thalamic hyperdensity (EI: 5, LI: 1), three of these also showed hyperdensity along the CSTs and cerebellum. Two of them showed additional hyperdensity of basal ganglia. One adult onset MRI also showed hyperdensity along the optic radiations [Figure 8].

### Serial MRI

**Figure 9:**
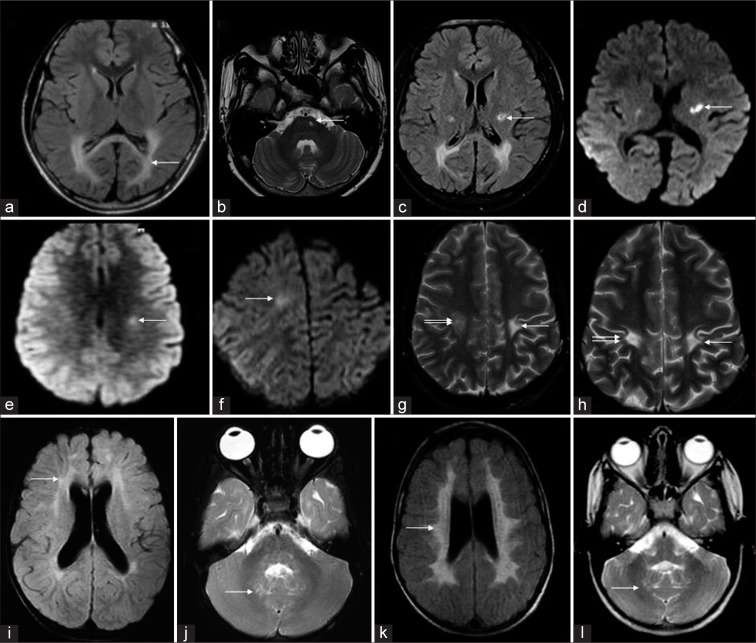
Temporal evolution of different cases of Krabbe disease on serial magnetic resonance imaging (MRI). A 6-year-old boy with Juvenile Krabbe (a-d) - initial fluid-attenuated inversion recovery (FLAIR) axial image (a) shows confluent posterior periventricular and splenial hyperintensity (white arrow). T2 axial (b), FLAIR axial (c), and diffusion-weighted imaging (DWI) (d) images from subsequent MRI done after 3 years show asymmetric CST involvement with bilateral restricted diffusion(arrows). A 14-year-old boy who presented initially with right hemiparesis which progressed to spastic quadriparesis over a 3-year period. Serial MRI (e-h) shows initial DWI with mild left CST restricted diffusion. Subsequent MRI (f) shows a new area of restricted diffusion along right CST. T2 axial (g) acquired at the same time shows chronic changes on the left side and more acute changes on the right. Later, MRI shows bilateral chronic changes along CST (h). Serial MRI (i-l) of a 5-year-old girl from juvenile group shows patchy scattered white matter changes on initial FLAIR axial image (i) progressing to confluent involvement with tigroid pattern in a subsequent MRI done after 34 months (k). Both scans (j, l) show dentate hilum involvement with sparing of deep cerebellar white matter.

Serial MRI was available in 8 children [Figure 9]. The range of interscan interval was 6–48 months with a mean of 17.7 months.

Three patients with pattern 1 showed minor interval changes with mild progression of extent and degree of white matter changes on subsequent imaging. In both pattern 2 and 3 new findings of signal changes and restricted diffusion was seen along the CSTs mostly in asymmetric distribution and in tandem on serial imaging. One child from juvenile group (pattern 4) had an unusual finding of patchy scattered white matter changes progressing to confluent involvement with tigroid pattern in a subsequent MRI done after 34 months. While both scans showed dentate hilum involvement, interestingly cerebellar white matter was spared even in the latter scan.

Thus, all early and late infantile subgroups in our series showed diffuse supra- and infra-tentorial white matter involvement with additional involvement of gray matter structures and cranial nerves. Adolescent and adult groups showed preferential isolated involvement of the CSTs and posterior deep white matter. Although juvenile group demonstrated heterogeneous cross-over patterns including thalamic and dentate hilum involvement, deep cerebellar white matter, basal ganglia and cranial nerves were notably spared.

## DISCUSSION

Dr. Krabbe in 1916 described a cohort of infants with a similar clinical presentation and autopsy findings.^[4]^ Deficiency of the lysosomal enzyme galactocerebrosidase in this disorder was discovered by Suzuki and Suzuki in 1970.^[5]^ Molecular cloning of the causative gene was done in 1993, and the first human mutation was reported in 1994.^[6,7]^ Since then, several case reports and series expanded the clinicoradiological spectrum. Primary pathology is of demyelination and apoptosis due to the accumulation of globoid cells and psychosine in the white matter.

Krabbe disease has been classified as early onset (< 6 months) and later onset (>6 months) variants. Loonen *et al.* further subclassified the late-onset phenotypes into late infantile (onset from 7 months to 3 years), juvenile (onset from 3 to 8 years), and adult (onset after 9 years) types.^[3]^ Significant clustering of clinical and radiological findings was noted among subtypes in our series when the latter classification was used. Early onset variants present with a relentless course with progressive loss of acquired milestones with spasticity, seizures, visual and mental deterioration, and periods of hyperpyrexia followed by eventual decerebrate posturing and succumb by 2 years of age. Late infantile onset variants are a continuum of the EI presentation, whereas juvenile and adult-onset forms present with slowly progressive spastic hemiparesis or quadriparesis, progressive visual loss, ataxia, neuropathy, and dementia with variable progression of symptoms and periods of stability sometimes lasting for years.^[1,8]^

Multiple studies have looked into correlation of clinical, biochemical, and genotypic variations. Homozygous 30 kb deletion though not highly prevalent has been found to be associated with early onset phenotype in Caucasian population, whereas p.G286D has been reported frequently in late-onset phenotype.^[9-11]^ Japanese studies have also put forward genotype-based predictions which may have significant implications in the management of asymptomatic patients.^[12]^ Pathogenic mutations in presaposin (PSAP) gene cause activator protein saposin deficiency leading to similar clinicoradiological phenotypes with normal enzyme levels.^[13]^ In our series, four children were confirmed with pathogenic mutations in GALC gene and two children had pathogenic mutations in PSAP gene with normal enzyme levels. Imaging of children with presaposin mutation was similar to the age-based clinical phenotype.

Distinct and consistent imaging patterns are known to be associated with different age-based subtypes of Krabbe disease. There is limited literature available on the systematic analysis of imaging spectrum of Krabbe, and largest series published until date is from Abdelhalim *et al.*^[14]^

EI group almost invariably shows signal changes of cerebellar white matter and dentate hilum giving a sandwiched appearance of the spared dentate nucleus.^[15]^ This is easily recognizable even in the 1^st^ month of life and should not be misinterpreted as ongoing myelination. Pyramidal tract signal changes are easily recognizable while supratentorial white matter abnormalities maybe obscured by ongoing myelination. All children in our cohort belonging to EI group consistently had deep cerebral and cerebellar white matter involvement, while this was around 53–85% in the cohort reported by Abdelhalim *et al.*^[14]^ Rest of the findings were comparable in our series. Supratentorial white matter abnormalities show central predominance with sparing of U fibers. Tigroid pattern was more common in our series than previously described.^[14]^ Trilaminar pattern was seen along PLIC in many of our cases, a feature which has not been highlighted in previous studies and can potentially mimic hypomyelination of early myelinating structures.

Thalamic volume loss with T2 hypointensity and optic nerve hypertrophy were characteristic findings of early-onset groups in our series as well. The cause for preferential enlargement of the prechiasmatic region and occurrence in this age group is not known. Atrophy of the orbital segments has also been reported due to psychosine-induced damage but was not appreciated in our cohort.^[16]^ Thickening and enhancement of cranial and spinal nerves are other exclusive findings of this group.^[17]^ Extensive cauda equina enhancement has also been described in previous reports but was not seen in our series.^[18,19]^ One child from late infantile group showed moderate patchy enhancement of the deep white matter. Although typically absent, very subtle enhancement has been described previously.^[20]^ One case of this subgroup showed enlarged medulla and cervical cord as previously described in literature.^[21]^ Remarkable diffuse cerebral and brainstem atrophy^[22]^ was also exclusively seen in the early-onset group.

Accurate comparison of other subgroups with previous studies is difficult due to the different criteria of age subgrouping used. A late infantile group has been shown to have heterogeneous and cross-over imaging patterns.^[14]^ Even though the upper limit of age at disease onset is 12 months in some of these studies as opposed to 36 months in our series, all four MRIs in our series had similar findings as the EI group except for lesser occurrence of optic nerve hypertrophy.^[14]^ This could be due to limited numbers or longer disease duration (12–24 months) at MRI. In the largest cohort of imaging described by Abdelhalim *et al.*,^[14]^ the occurrence of dentate nucleus and cerebellar white matter signal changes was variable in late infantile group.

Adult and juvenile subgroups characteristically show the involvement of CSTs, callosal splenium, and posterior periventricular and deep white matter. Different permutations and combinations of involvement of these structures were observed in our cohort. The variable presentations and imaging features in this late-onset group suggest that there are additional subphenotypes that may not fall neatly into age-based categories or imaged-based patterns. Subcortical and frontal involvement described in later stages of disease was absent in our series. Similar to previous reports, the adult subgroup in our series showed sparing of cerebellar white matter, dentate hilum and thalamus as well as absence of diffuse cerebral and cerebellar volume loss. Our study showed asymmetry of CST involvement in 70% of MRIs. Symmetrical/asymmetrical and even unilateral patterns have been described previously.^[20]^ On serial imaging, there was restricted diffusion involving the side corresponding to the clinical symptomatology.

While early-onset and late-onset groups showed distinct patterns, juvenile form was the most heterogenous and showed the maximum numbers of different pattern types. One novel pattern which was seen only in this group was of patchy supratentorial white matter hyperintensities with isolated dentate hilum hyperintensity without deep cerebellar white matter signal changes. One of these cases on subsequent scan developed confluent supratentorial white matter signal changes with tigroid appearance without cerebellar white matter involvement. Thalamic involvement in the form of volume loss and heterogeneous hyperintense or hypointense signal changes were also seen in the juvenile group, though the clinical implications are not known.

MRS may show reduced N-acetyl aspartate and elevation in choline and myoinositol peaks.^[23]^ DTI has been reported as an *in vivo* biomarker of microstructural damage in normal appearing white matter in asymptomatic neonates and has a probable role in post-transplant prognosis and response assessment.^[24]^ In our series, DTI was not available in the early-onset age group. One case from late-onset group showed reduced anisotropy in involved areas.

CT often shows hyperdensity of thalami, brainstem, cerebellum, basal ganglia, and CSTs in EI group. A previously unreported finding of hyperdensity of optic radiations was noted in one adult onset patient. When supratentorial white matter signal changes are not well recognizable from ongoing myelination on MRI, CT may contribute to the diagnosis.

**Figure 10:**
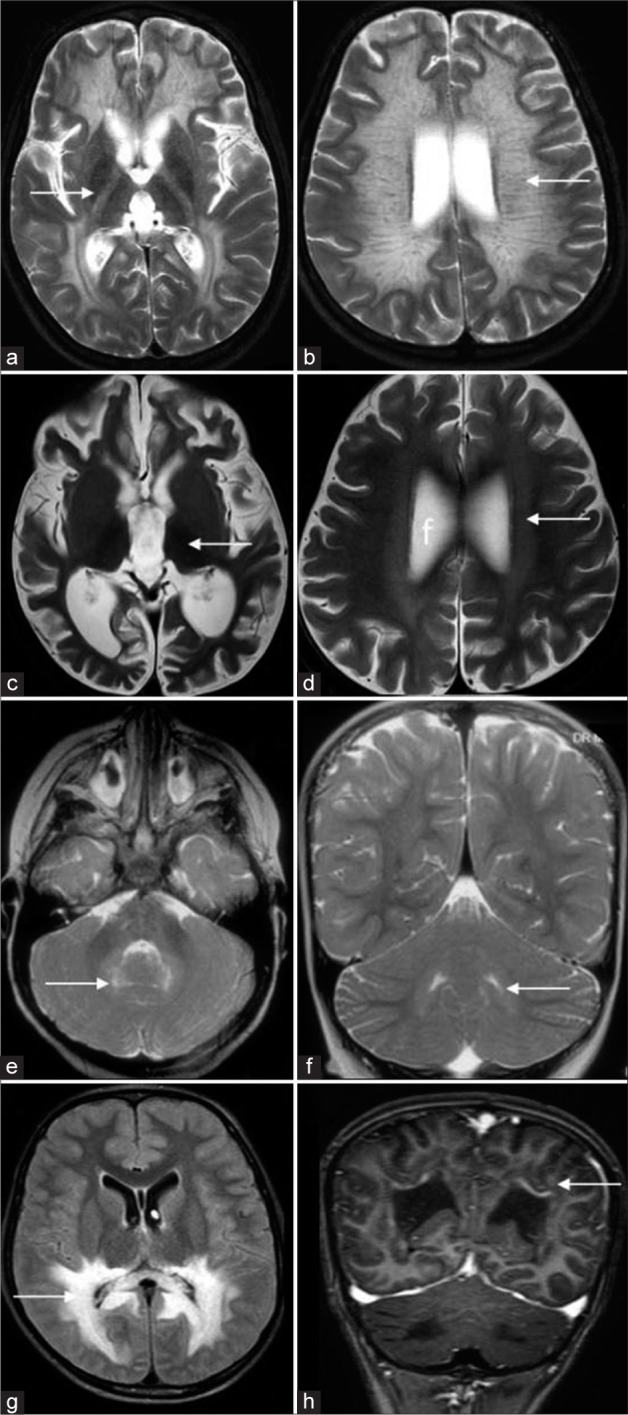
Differential diagnosis of Krabbe disease. T2 axial (a, b) of a case of metachromatic leukodystrophy shows diffuse white matter hyperintensity (white arrow in a) with posterior limb of internal capsule involvement and tigroid appearance (white arrow in b). T2 axial images (c, d) of a case of neuronal ceroid lipofuscinosis show diffuse thalamic volume loss and hypo intensity (white arrow in a) and subtle white matter hyperintensity (white arrow in b). T2 axial and coronal images (e, f) of peroxisome biogenesis disorder show dentate hilum hyperintensity (white arrows). Fluid-attenuated inversion recovery axial and T1 post-contrast coronal images of adrenoleukodystrophy (g, h) show confluent posterior periventricular and callosal splenial hyperintensity (white arrow in a) with enhancement of the intermediate zone (white arrow in b).

Loes devised a 0–32-point scoring system based on location and extent of disease and the presence of atrophy.^[25]^ The scoring system is more valuable in the objective follow-up of Krabbe disease patients undergoing hematopoietic stem cell transplantation (HSCT) than in initial diagnosis. Many disorders can mimic both early- and late-onset Krabbe disease clinically and radiologically. A comprehensive list has been tabulated [Table 3 and Figure 10].

**Table 3:** Differential diagnosis of Krabbe disease.

Disorder	Clinical features	Supratentorial white matter	Infratentorial white matter	Deep grey matter	Cerebral and cerebellar volume loss	Others/Comments
Early onset Krabbe disease	Relentlessly progressive spasticity, visual and hearing loss, seizures, decerebrate rigidity, unexplained hyperpyrexia	Periventricular and deep white matter with subcortical sparing, tigroid pattern	Pyramidal tracts Cerebellar white matter and dentate hilum typically involved	Thalamic hypointensity is typical	Marked cerebral volume loss	CT hyperdensity of thalamus Optic nerve hypertrophy Cranial nerve enhancement usually seen
Metachromatic leukodystrophy	Progressive motor and cognitive regression, sensory-motor demyelinating neuropathy, visual and hearing loss, seizures	Periventricular and deep white matter with subcortical sparing, tigroid pattern, Frontal predominance in late-onset	Pyramidal tracts Cerebellar white matter in some	Thalamic hypointensity may be seen	Less pronounced	CT hyperdensity is absent Enhancement of cranial nerves and spinal roots may be seen
Peroxisome Biogenesis Disorders	Dysmorphic facies, hypotonia, psychomotor retardation, hepatomegaly, sensorineural hearing loss, pigmentary retinopathy	Variable, commonly corticospinal tracts and posterior deep white matter, callosal splenium	Deep cerebellar white matter and dentate hilum	Absent	Can be present	Cortical malformations can be associated Some patterns can mimic adult-onset Krabbe as well
Hypomyelination of early myelinating structures	Normal neonatal period and early development followed by developmental stagnation, nystagmus, spasticity, and cerebellar ataxia	Trilaminar or tram-track PLIC, Periventricular and parietal white matter/optic radiations	Absent/mild peridentate white matter hyperintensity Hyperintensity of pons, medulla	Absent	Absent	
ITPA mutation related early infantile epileptic encephalopathy	Progressive microcephaly, refractory seizures with onset in early infancy, profound developmental disability, cataract, cardiac conduction defects	T2 hyperintensity and restricted diffusion of PLIC Delayed myelination Variable restricted diffusion of optic radiations	Variable pyramidal tracts in the midbrain, middle and inferior cerebellar peduncles, dentate hilum and cerebellar white matter hyperintensity and restricted diffusion	Absent	Progressive cerebral atrophy	Serial MRIs may show less conspicuous T2 and DWI signal changes of PLIC
Neuronal ceroid lipofuscinosis	Visual loss due to maculopathy, regression of motor and intellectual functions, myoclonic seizures, ataxia and spasticity	Mild long TR hyperintensity of white matter, PLIC involvement is late except in LI variant	Usually spared	Marked thalamic T2 hypointensity and volume loss in later stages	Pronounced volume loss Cerebellar >Cerebral in LI variant	
Mitochondrial disorders	Heterogeneous group with recurrent encephalopathy, ataxia, spasticity, seizures, neuropathy, visual loss, cardiac and liver involvement	Confluent deep white matter hyperintensity with or without restricted diffusion	Variably involved	Basal ganglia and thalamus can be involved depending on the specific gene involved	Can be present	Significant Restricted diffusion is usually present and is a distinguishing feature
Krabbe disease later onset	Progressive spastic hemiparesis or quadriparesis, progressive visual loss, ataxia, neuropathy and cognitive decline	Posterior periventricular, splenial and CST involvement No zonal pattern or enhancement	Absent	Absent	Absent	No enhancement diffusion restriction can be seen
Adrenoleukodystrophy/adrenomyeloneuropathy (AMN)	Progressive visual and hearing loss, cognitive decline with behavioral disturbances, spasticity, and ataxia. AMN presents in third-fourth decade with slowly progressive spasticity, neuropathy, bladder and bowel incontinence	Posterior periventricular and splenial with CST involvement Zonal pattern with enhancement and restricted diffusion of leading edge	Absent	Absent	Absent	
ALS	Progressive asymmetric muscle weakness of limbs, fasciculations, UMN signs, bulbar weakness, and breathlessness	Isolated corticospinal tract involvement with milder T2 hyperintensity	CST	Absent	Absent	T2 hyperintensity is usually milder than seen in Krabbe disease

PLIC: Posterior limb of internal capsule, CST: Corticospinal tract, MRI: Magnetic resonance imaging, DWI: Diffusion-weighted imaging, LI: Late-infantile

HSCT if performed for the EI phenotype before the infant reaches 2 months of age has been shown to prevent the disease manifestations and has become the standard of care in developed countries.^[26,27]^ There are anecdotal reports of arrest of disease progression in asymptomatic/mildly symptomatic patients in late-onset subgroup as well.^[28]^ However, considering their phenotypic variability, interpretation of the results is difficult and is usually offered on an individual basis.

### Prediction of clinical progression and treatment response by imaging – An area of growing need

Given the poor correlation with both enzymatic activity and genotype, management of asymptomatic infants diagnosed by newborn screening remains a major conundrum.^[29-31]^ Heterogeneous and unpredictable clinical course in later onset subgroups is another management dilemma. In this context, the possible role of imaging in predicting disease course becomes very crucial. While this has not been extensively evaluated, several suggestions have evolved from previous studies. It has been found that early involvement cerebellar white matter and dentate hilum in late infantile age subgroup predict rapidly deteriorating disease course.^[14]^ It has been shown that MRI may be abnormal in asymptomatic children and this has a huge implication in prognostication and management decisions. Longitudinal studies on deep MRI phenotyping including volumetry, parcellation, spectroscopy, and advanced microstructural imaging techniques, especially, in pre and early symptomatic cases may contribute toward identifying the role of imaging in phenotype prediction. This may also bring more insight into the pathophysiology of selective vulnerability of different structures at different ages and explain the imaging and clinical heterogeneity associated with the same genotype.

### Limitations

Retrospective nature and inclusion of only symptomatic cases were the main limitations. Hence, temporal relation of symptom onset and imaging abnormality could not be assessed. The absence of genetic data in most cases precluded correlation analysis with MR phenotype. None of the children underwent HSCT in this series, limiting assessment of the evolution of imaging post treatment. Non-availability of advanced imaging in all cases was another major limitation. Clinical course and prognosis were not correlated with imaging features in this study.

## CONCLUSION

Awareness of distinct neuroimaging findings in early- and late-onset Krabbe disease allows timely diagnosis, guide management decisions, and genetic counseling. Exploring the role of MRI including advanced imaging techniques as a biomarker of outcome prediction can potentially resolve some of the persisting therapeutic uncertainties. Given the rarity of the disease and clinicoradiological heterogeneity, this will be possible only by actively fostering international collaborations.
